# Future Trends in Semiconducting Gas-Selective Sensing Probes for Skin Diagnostics

**DOI:** 10.3390/s21227554

**Published:** 2021-11-13

**Authors:** Anthony Annerino, Pelagia-Irene (Perena) Gouma

**Affiliations:** Department of Materials Science and Engineering (MSE), The Ohio State University, Columbus, OH 43210, USA; Annerino.2@osu.edu

**Keywords:** biomarkers, breath, skin gas, diagnostics, monitoring

## Abstract

This paper presents sensor nanotechnologies that can be used for the skin-based gas “smelling” of disease. Skin testing may provide rapid and reliable results, using specific “fingerprints” or unique patterns for a variety of diseases and conditions. These can include metabolic diseases, such as diabetes and cholesterol-induced heart disease; neurological diseases, such as Alzheimer’s and Parkinson’s; quality of life conditions, such as obesity and sleep apnea; pulmonary diseases, such as cystic fibrosis, asthma, and chronic obstructive pulmonary disease; gastrointestinal tract diseases, such as irritable bowel syndrome and colitis; cancers, such as breast, lung, pancreatic, and colon cancers; infectious diseases, such as the flu and COVID-19; as well as diseases commonly found in ICU patients, such as urinary tract infections, pneumonia, and infections of the blood stream. Focusing on the most common gaseous biomarkers in breath and skin, which is nitric oxide and carbon monoxide, and certain abundant volatile organic compounds (acetone, isoprene, ammonia, alcohols, sulfides), it is argued here that effective discrimination between the diseases mentioned above is possible, by capturing the relative sensor output signals from the detection of each of these biomarkers and identifying the distinct breath print for each disease.

## 1. Introduction

Disease diagnosis based on the human body’s gaseous emissions can be traced back as far as Hippocrates of Cos, who lived between 460 BCE and 370 BCE and who is commonly known as humanity’s first physician. Hippocrates was known to smell patients’ breath and various body parts to determine his recommended treatment [1]. Even medical terms dating back this far also still exist today, such as “fetor hepaticus”, referring to the sweet breath accompanying late-stage liver failure, which we now know is caused by the relative abundance of ketones in breath resulting from liver failure. Despite evidence that analytes appearing in breath and ambiently coming out of the skin correlate with various diseases, breath and skin gas diagnostics is not common among modern physicians. Such noninvasive disease detection stands to dramatically raise the quality of life of people around the world, and ceramic sensor nanotechnology is the driving force at the heart of recent advances in this field [2]. NO, CO, and many VOCs are known biomarkers that are products of central biological processes and scale in concentration depending on the state of one’s health. Lung inflammation is widely correlated with NO concentration in breath. Various cardiovascular and metabolic diseases such as nephritis and diabetes influence CO concentration in breath [3]. Isoprene is known to relate to cholesterol levels, and acetone concentration in breath changes more acutely with insulin levels than blood glucose concentrations [4,5]. It is then obvious that an array of quantitative single-gas sensors targeting biomarkers established to be emitted from the skin could simultaneously check for a litany of issues or even continuously monitor patients’ total health.

It is critical to understand that such a device is not an electronic nose that simply searches for random patterns that pattern-recognition algorithms correlate with observed conditions. It is possible to make individual sensors with established sensitivities for specific analytes that have been proven to scale with the severity of certain diseases. Such quantitative measurements would allow not just binary readings such as “ill” versus “well”, but would allow indirect measurement of biologically significant quantities such as blood sugar. This is not to say that electronic noses are without merit because there are several significant examples demonstrating that electronic noses have potential utility in diagnosing issues such as urinary tract infections and even possibly certain cancers [6,7,8,9]. The other major distinguishing factor separating devices foreseen here and the electronic noses just mentioned is that those electronic nose devices are meant for momentary diagnosis from a sample actively provided by a patient such as a urine sample, not continuous monitoring of the gases ambiently being emitted from the patient’s skin.

Researchers have collected gases emitted from human skin from areas such as the arms, hands, and fingers and have reported their relative values as measured using gas chromatography and/or mass spectrometry [10,11]. Most correlate with their relative concentrations in breath and blood. For acetone from skin, the normal concentration is about 80 ppb and has been measured to be as much as over 1 ppm in subjects undergoing ketosis. Ammonia and isoprene concentrations emitted from skin vary within a few to tens of ppbs normally, but it should be noted that the technologies commonly used to measure them do not have the accuracy of resistive chemosensing. Thus, highly selective and sensitive resistive sensing probes may be able to detect all of these gases of interest. Furthermore, skin prints of different diseases may be inferred based on the analysis of the headspace above the skin. Finally, skin gas is cleaner than breath since it is essentially filtered by the dermal layer, and skin gas is released continuously in amounts controlled by the autonomic nervous system. These are just a few of the added advantages to developing skin gas detectors.

## 2. The State-of-the-Art Biomarkers

Great barriers to widespread acceptance of breath and skin-emission sensing technologies, besides alcohol detection in the determination of intoxication, still exist, along the path of the professional adoption of these techniques and along the path of public trust of these techniques. Most notable among these barriers is the disagreement among health professionals on where to draw the line between healthy and ill in terms of gaseous metabolite biomarker readings, but efforts by professional organizations such as the American Thoracic Society and the European Respiratory Society to rigorously determine such guidelines are inspiring new efforts to develop user-friendly breath-monitoring devices for widespread public use [12]. Within this context, we should explore sensor arrays targeting, for example, NO, CO, and sulfides, as potential biomarkers for cystic fibrosis. In a strategy that is fundamentally different from the electronic olfactory systems assembling arrays of nonselective sensors, the so-called electronic nose devices, this approach involves the use of a few sensing elements uniquely sensitive to individual chemical species set up as a relatively simple sensory system, more similar to those that are well established in nonhealth applications. Correlating the individual sensing responses would then allow mapping out the response space and identifying unique response signatures belonging to distinct health issues ranging from the early indicators of cystic fibrosis to one’s blood sugar.

Therefore, the right sensor array could allow for the diagnosis and monitoring of an enormous range of diseases and conditions, ranging from asthma and cystic fibrosis to flu and COVID-19, to Alzheimer’s and different cancers. This would ideally be a wearable device for continuous monitoring. Of course, the main challenge on the level of the materials’ development is the design of sensor materials with sensitivity limits below the incredibly low concentrations of these biomarkers, and the sensing elements inside such a device may take on morphologies such as nanowires and nanogrids, as described below, because of the various advantages offered by these morphologies over more traditional morphologies.

Skin monitors have been shown to work with sweat over long-term use; the prime example is the Secure Continuous Remote Alcohol Monitoring System (SCRAM) [13]. One of the very few fully developed continuous skin monitors, the SCRAM, is a sensor in an ankle bracelet, which tests the sweat and gas over the skin and transmits data through radio frequency to a modem to measure transdermal alcohol contact. However limited skin chemical sensor technology is, skin gas sensor technology is even more limited. Hardly any fully developed, portable, stand-alone technology exists for skin gas sensing in the absence of sweat.

Notably, previous works have established a direct correlation between acetone released from skin (e.g., hands, arms, fingers) and acetone exhaled with breath [14], which, in turn, has a direct correlation with the metabolic rate [15]. Indeed, clinical studies with diabetic patients reported the ***concentration of skin acetone to correlate with breath acetone*** and blood β-hydroxybutyrate, while studies investigating the influence of cycle exercise on acetone showed that the skin gas acetone concentration is “significantly related” to the acetone concentration in breath [16,17,18]. One skin acetone sensor made of a zeolite array has been developed, but it was tested only for sensitivity, and not for selectivity or any other properties that must be determined for skin gas applications [19].

## 3. Ceramic Semiconducting Sensing Probes

Ceramic gas sensors have found applications all around us since Taguchi’s first commercial resistive sensor came to public attention in 1968, not least of all because of their low cost [20]. Resistive sensors function on the principle that interactions such as adsorption of gaseous species change the electrical resistance of the sensing material, and the magnitude of this change correlates quantitatively with the concentration of the analyte in the headspace around the sensor. Metal-oxide-based ceramics are widely used to detect O_2_ in vehicles, alcohol in breath, CO in buildings, etc. All sensor testing mentioned here was conducted with a two-probe resistance-measuring method using a digital multimeter where the sensor material was deposited atop interdigitated electrodes prepatterned on alumina substrates.

Given their distinct morphology, metal oxide nanowires have seen a boom in popularity because of their high surface-area-to-volume ratio, which allows more interaction with the headspace per unit volume of the sensor material, thus increasing sensor sensitivity. Beyond this, single-crystal nanowires have shown extra promise because the careful growth of single crystals allows one to choose which crystal planes are exposed at the nanowire surface, and it is well established that certain crystal systems exhibit drastically different gas sensitivity and/or selectivity along different crystallographic planes. Additionally, single crystals have been shown to exhibit more perfect adherence to stoichiometry than polycrystals, which is beneficial for the stability of the already fragile nanowire structure.

### 3.1. Nanowires of α-MoO_3_ for Selective Ammonia Sensing

**Figure 1:**
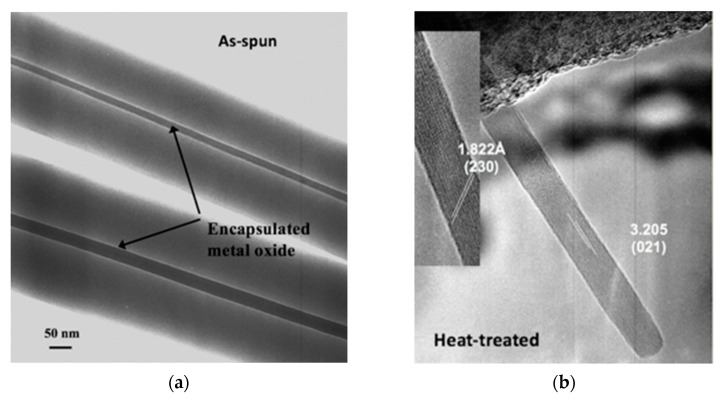
(**a**) As-spun fibers [21]; (**b**) post-heat-treatment α-MoO_3_ nanowires [21].

One study involved the use of electrospinning for the fabrication of α-MoO_3_ nanowires for NH_3_ sensing [21]. It was shown that single crystal α-MoO_3_ nanowires could be made from a molybdenum isopropoxide sol in the two procedurally simple steps of electrospinning a mixture of the sol and a polyvinylpyrrolidone and calcining the electrospun mats at 500 °C to burn off the organics and leave behind the metal oxide. Electron microscopy studies elucidated that these nanowires ranged between 10 nm and 15 in diameter and between 1 µm and 2 µm in length. The ability of the electrospun single crystal nanowires to function as a resistive sensor was tested against that of nanocrystalline films made from a sol–gel of the same precursor material calcined according to the same heat treatment procedure. Sensing experiments conducted at 450 °C after 8 h of equilibration at 500 °C found that the electrospun single-crystal nanowires exhibited double the sensitivity of the film upon exposure to 50 ppm NH_3_ in an 80:20 mixture of N_2_ and O_2_, and the superiority of the electrospun single-crystal nanowires became even more obvious with every increase in concentration. At 300 ppm NH_3_, the highest concentration tested, the sensitivity of the electrospun single-crystal nanowires was an order of magnitude higher than that of the sol–gel film of the same material. Subsequently, there is reason to expect that metal oxides prepared in this way may be measurably sensitive in the range of a few parts per billion, the concentration range of NH_3_ ambiently emitted from the skin. Figure 1 displays images of these electrospun nanowires in the as-spun condition where the bulk of the volume is still polymer and in the heat-treated condition where the polymer has been burned off and all that remains is the α-MoO_3_.

### 3.2. CuO Nanowires for H_2_S Detection

**Figure 2:**
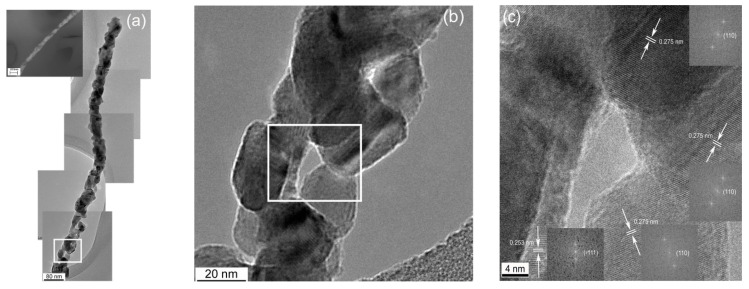
(**a**) Stitched HRTEM image of a polycrystalline CuO nanofiber; (**b**) enlarged section of the CuO nanowire showing particle morphologies; (**c**) enlarged subsection of the CuO nanowire showing the relative particle orientations [30].

Irritable bowel syndrome (IBS), a digestive sickness without a single well-known cause, is closely associated with many intestinal problems including diarrhea, constipation, bloating, and sometimes malabsorption and malnutrition [22,23,24]. An IBS diagnosis presently requires endoscopy or other invasive testing, but it was discovered not long ago that small intestinal bacterial overgrowth (SIBO) strongly correlates with IBS. Consequently, new life came to efforts to devise noninvasive methods for indirectly evaluating SIBO. It appeared that the concentration of hydrogen in breath following digestion of lactulose and/or glucose is related to the likelihood of SIBO [25]. Unfortunately, this hydrogen breath test for diagnosing SIBO could not be accurately standardized, and more recent developments suggest that the validity of breath testing is dubious in this application [26,27,28]. It has since been found that the concentration of H_2_S in breath scales more reliably as a function of SIBO and severity of diarrhea-predominant IBS. H_2_S can be detected at trace levels with CuO, a p-type semiconductor. In another work, a CuO nanogrid sensor with a continuous, self-supported, intertwined nanograin architecture offering an extremely high surface area and interconnected porosity was also fabricated by electrospinning [29]. In this case, a solution of only PVP was electrospun onto a Cu mesh, and the whole assembly was heat treated at 500 °C. Figure 2 displays images of the multicrystal CuO nanowires grown from the Cu mesh during the heat treatment [30]. While no H_2_S-sensing tests have been conducted with this material in this morphology yet, CuO nanowires have been established as a successful H_2_S-sensing material by others [28].

### 3.3. Nanowires of Hexagonal WO_3_ for Selective Isoprene Detection

**Figure 3:**
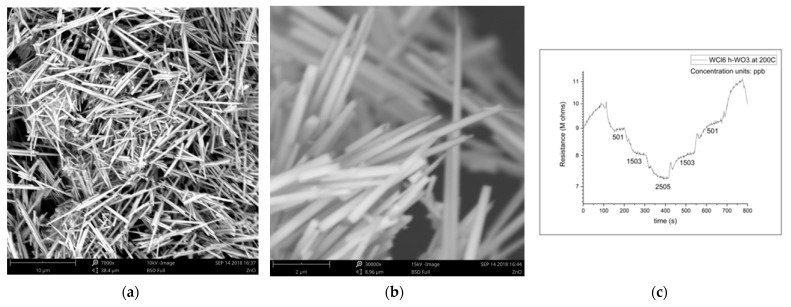
(**a**) h-WO_3_ nanowires; (**b**) h-WO_3_ nanowires; (**c**) h-WO_3_ nanowire isoprene-sensing results [31].

**Figure 4:**
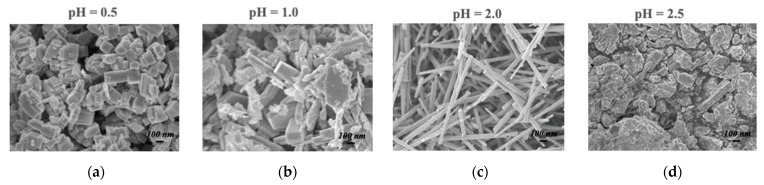
Hydrothermally synthesized h-WO_3_ from: (**a**) a starting pH of 0.5; (**b**) pH 1.0; (**c**) pH 2.0; (**d**) pH 2.5 [31].

The magnitude of the sensing response, recovery time, and detection limit are all established advantages of the nanowire morphology in resistive gas sensing, so it is no surprise that h-WO_3_ nanowires outperform conventionally fabricated h-WO_3_ in sensing isoprene, another important gaseous biomarker. This improvement is expected to enable quick-pass scanning of the skin gas headspace despite the low concentrations available for detection. Figure 3 illustrates the morphology and dimensions of h-WO_3_ nanowires fabricated by hydrothermal synthesis and the sensing response of these nanowires under the exposure of ppb of isoprene in a balance of breathing grade air at 200 °C [31]. Hydrothermal synthesis is renowned for its utility in synthesizing metastable metal oxide phases, and the morphology of the produced particles can be easily toggled via the pH of the starting solution, as displayed in Figure 4.

### 3.4. Ferroelectric ε-WO_3_ for Acetone Sensing

Ferroelectric poling has been shown to drive selectivity, which ultimately reduces the need for complex machine-learning routines by reducing the number of possible interferants [31,32,33,34,35,36]. The most important interferants to be able to differentiate from the intended analyte are ones with chemical structures similar to that of the analyte, and other important interferants to be aware of are the other species known to be emitted from the skin. Table 1 demonstrates this selectivity of 10% Cr-doped ε-WO_3_ sensors on the basis of the analyte’s dipole moment [36]. Such a material was fabricated by flame spray pyrolysis from a solution of ammonium tungstate hydrate and chromium (III) acetylacetonate in diethylene glycol and ethanol, and the sensor testing was carried out at 400 °C in a balance of air.

## 4. Conclusions

The medical community has been aware of the diagnostic potential of the gaseous metabolites emitted from the human body for centuries; however, even with the recent technological advances, this potential is nowhere near fully realized. Skin-gas-testing devices using the technologies described here and others envisioned that are not yet ready to be reported are expected to work as magic wands, passing over one’s arm and reading the gaseous headspace above the skin for disease in real time, creating a new era for point-of-care health screening. The conditions most readily monitored or diagnosed by such skin-emission sensing include kidney failure, influenza, irritable bowel syndrome, and diabetes via ammonia, isoprene, H_2_S, and acetone sensing, respectively. Such monitoring will enable new levels of health awareness by allowing real-time quantitative tracking of gaseous biomarkers continuously emitted from the skin, which cannot be offered by devices meant simply to notice chemical patterns in the headspace above the skin.

## Figures and Tables

**Table 1:** e-WO_3_ sensing results [36]. Reprinted with permission from L. Wang, A. Teleki, S.E. Pratsinis, and P.I. Gouma, “Ferroelectric WO_3_ Nanoparticles for Acetone Selective Detection”, *Chem. Mater*., 20(15), pp. 4794–4796, 2008. Copyright 2021 American Chemical Society.

Analyte	Dipole Moment (D)	Sensitivity at 0.2 ppm	Sensitivity at 0.5 ppm	Sensitivity at 1 ppm
Acetone	2.88	1.55	2.05	2.90
Ethanol	1.69	1.08	1.15	1.32
Methanol	1.70	1.03	1.10	1.23
NO	0.159	1.00	1.05	1.09
NO_2_	0.316	1.00	1.04	1.07
NH_3_	1.417	1.02	1.03	1.05
CO	0.112	1.00	1.00	1.00

## Data Availability

Access to Data is available as indicated by the sponsor guidelines.
